# Hourglass urinary bladder in a spinal cord injury patient - unusual late complication of suprapubic cystostomy: a case report

**DOI:** 10.1186/1757-1626-2-6866

**Published:** 2009-05-18

**Authors:** Subramanian Vaidyanathan, Peter L Hughes, Bakul M Soni, Gurpreet Singh, Paul Mansour

**Affiliations:** 1 Regional Spinal Injuries Centre, District General HospitalSouthport PR8 6PNUK; 2 Department of Radiology, District General HospitalSouthport PR8 6PNUK; 3 Department of Urology, District General HospitalSouthport PR8 6PNUK; 4 Department of Cellular Pathology, District General HospitalSouthport PR8 6PNUK

## Abstract

**Introduction:**

Suprapubic cystostomy is performed in spinal cord injury patients in order to prevent complications associated with long-term urethral catheter drainage. We report a patient in whom suprapubic catheter did not drain urine satisfactorily and imaging studies revealed hourglass bladder.

**Case presentation:**

A female patient sustained paraplegia in a traffic accident in 1994 at the age of seventeen years. When she was discharged from spinal unit, she was performing self- catheterisations. In 1995, indwelling urethral catheter drainage was instituted, as she was not able to cope up with self-catheterisations. Intravenous urography, performed in 1994, 1997, 2000 and 2003 showed urinary bladder of normal shape. In 2004, this patient developed frequent blockages and bypassing of catheter; therefore, suprapubic cystostomy was performed. In 2005, she was leaking urine per urethra; therefore, an indwelling catheter was inserted; both suprapubic and urethral catheters drained urine. In 2008, suprapubic catheter failed to drain any urine. Cystogram revealed hourglass bladder. The balloon of suprapubic Foley catheter was located in the upper compartment of hourglass bladder whereas the urethral catheter was placed in the inferior compartment. Ultrasound examination of urinary bladder showed two compartments of hourglass bladder separated by a narrow waist. Computed tomography cystogram delineated smaller superior and larger inferior compartment of the hourglass bladder. At present this patient is happy to manage her bladder with suprapubic and urethral catheters.

**Conclusion:**

When prompt replacement of a mal-functioning suprapubic catheter fails to rectify the problem, computer tomography cystography should be performed to check precise location of suprapubic catheter and structural abnormalities of urinary bladder. In this patient, cystogram revealed hourglass bladder. Possible reasons for development of hourglass bladder in spinal cord injury patients are: traction applied to dome of urinary bladder by Foley balloon when suprapubic catheter is taped tightly to anterior abdominal wall for several months; uncoordinated contractions of detrusor muscle; chronic cystitis leading to hypertrophy of bladder wall.

## Introduction

Suprapubic cystostomy is recommended in spinal cord injury patients in order to prevent complications associated with long-term urethral Foley catheter drainage such as penile urethral split in male patients and erosion of bladder neck in female patients. Although suprapubic cystostomy is a safe procedure in the majority of cases, unusual complications may occur either immediately after suprapubic cystostomy [1,2] or a few years later. We report a female paraplegic patient in whom intravenous urography revealed normal bladder in 2003; suprapubic cystostomy was performed in 2004; Four years later, cystogram revealed hourglass bladder.

## Case presentation

**Figure 1.**
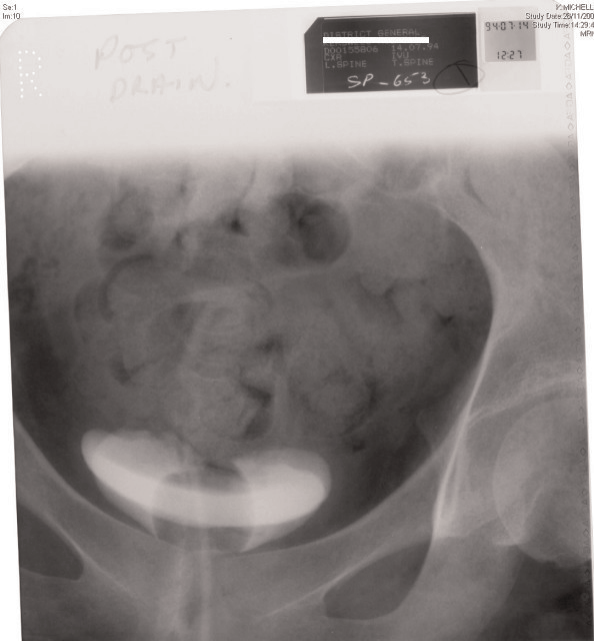
Intravenous urography, performed on 14 July 1994, shows urinary bladder, which is of normal contour. The balloon of Foley catheter can be seen.

**Figure 2.**
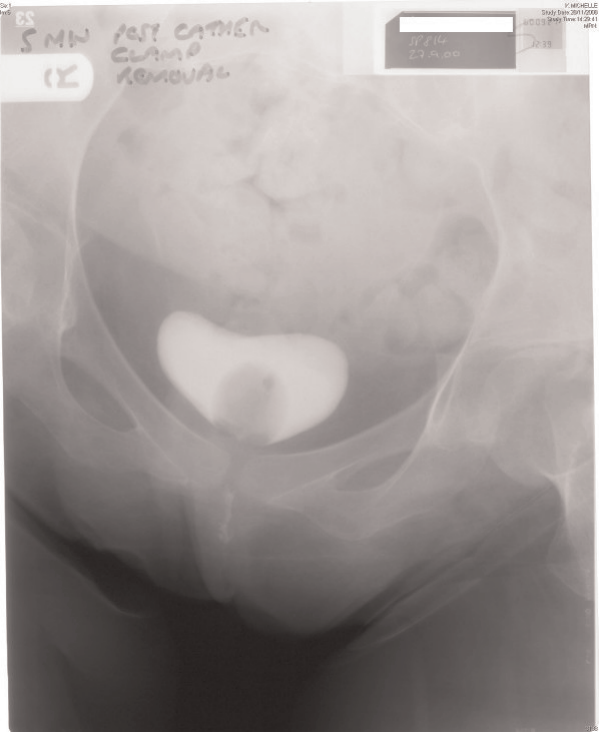
Intravenous urography, performed on 27 September 2000, shows urinary bladder, which is of normal contour. The balloon of Foley catheter can be seen.

A 31-year old Caucasian woman sustained T-12 complete paraplegia after a road traffic accident on 18 February 1994. She was riding her bicycle coming back from her mother's home when she was hit by a heavy goods vehicle and dragged under the lorry for about 120 yards. There was degloving injury with extensive soft tissue loss of right thigh, trauma to right femoral artery, fracture of lateral femoral condyle (right). She underwent vascular surgical repair of right femoral artery and plastic surgery for right thigh. Following transfer out of intensive care unit, she had MRI of dorso-lumbar spine. There was flattening of spinal cord from T-3 to T-7 by a small epidural haematoma. She had developed complete paraplegia at T-9 level. After undergoing rehabilitation, she was discharged home on 15 December 1994 and was managing neuropathic bladder by self-intermittent catheterisation, Intravenous urography, performed on 14 July 1994 (Figure 1), 02 April 1997, 27 September 2000 (Figure 2) and 08 October 2003 showed normal kidneys and normal bladder.

In 2004, this patient developed frequent blockages and bypassing of catheter; therefore, suprapubic cystostomy was performed on 26 March 2004. In January 2005, this patient attended spinal unit with history of suprapubic catheter not draining urine and she was leaking urine per urethra. Cystoscopy revealed that the suprapubic catheter was in proper place. There were no stones in the bladder. The urethra was patulous. A 14 French Foley catheter was inserted per urethra. Both suprapubic and urethral catheters were draining clear urine. This patient was happy to manage with urethral and suprapubic catheters, which were connected to separate bags and attached to right and left leg respectively. In August 2006, she was prescribed trospium chloride, 20 mg, twice daily. Video-urodynamics, performed on 21 November 2007, showed no detrusor overactivity. There was incontinence due to weakness of urethral sphincter. She was advised to consider following treatment options:

- Managing with two catheters
- Closure of urethra but this would risk making the bladder over-active.

This patient decided to continue with urethral (size 14 French) and a size 22 French suprapubic Foley catheter drainage. Both catheters were draining urine and there was very little urine leak per urethra.

**Figure 3.**
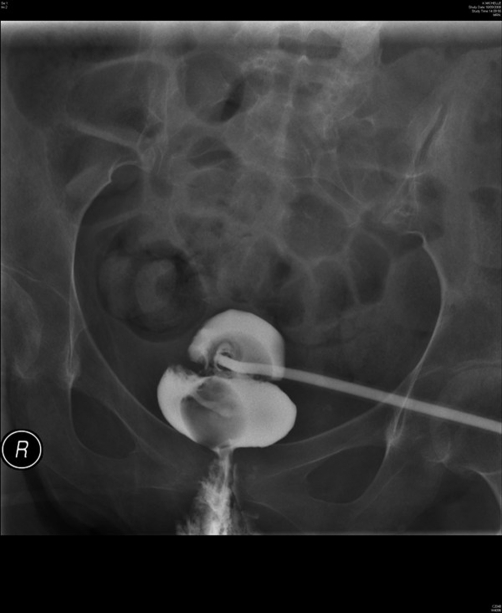
Cystography, performed on 18 September 2008, shows hourglass urinary bladder. The balloon of suprapubic Foley catheter is seen in the superior compartment of hourglass bladder whereas the balloon of urethral Foley catheter is located in the inferior compartment of hourglass bladder.

**Figure 4.**
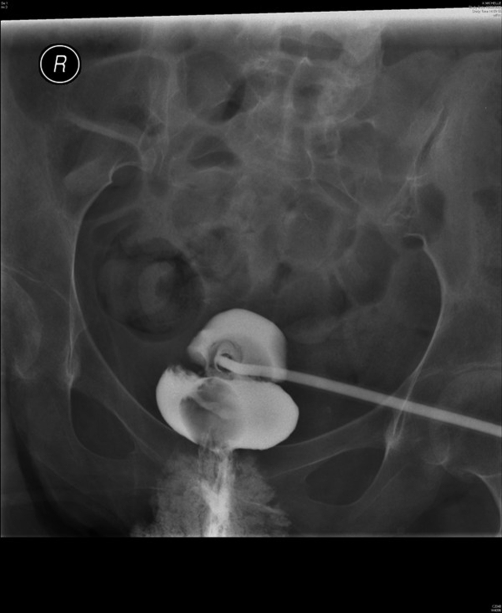
Cystography, performed on 18 September 2008, shows hourglass urinary bladder. The suprapubic Foley catheter is emerging from superior compartment of hourglass bladder. The balloon of urethral Foley catheter is located in the inferior compartment of hourglass bladder.

**Figure 5.**
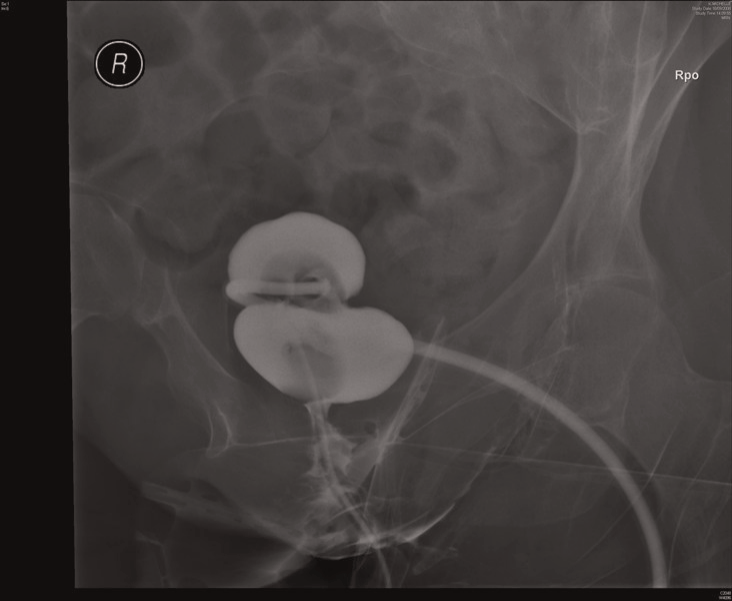
Cystography, performed on 18 September 2008, shows upper and lower compartments of hourglass urinary bladder very distinctly.

In September 2008, this lady attended spinal unit with history of suprapubic catheter not draining urine at all. The suprapubic catheter was flushed with sterile saline but it would not go in. The impression was blockage of suprapubic catheter. Therefore, the catheter was removed and a 22 French silicone Foley catheter was inserted. Surprisingly, the new catheter also failed to drain urine. This catheter was taken out and inspected; the catheter was found to be patent. Another catheter was inserted and cystogram was performed on 18 September 2008. Cystogram revealed hourglass bladder. The balloon of suprapubic Foley catheter was seen in the upper compartment and the urethral Foley catheter was located in the larger inferior compartment of hourglass bladder (Figures 3,4 and 5).

**Figure 6.**
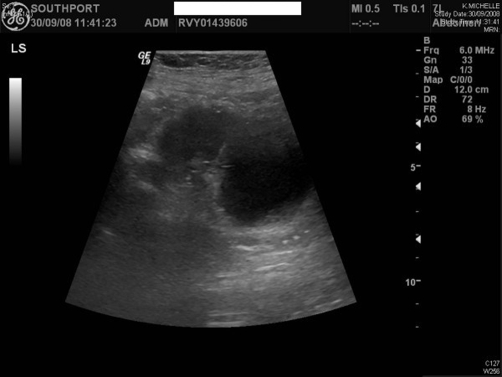
Sagittal ultrasound scan of urinary bladder, performed on 30 December 2008: The two compartments of hourglass bladder are seen.

**Figure 7.**
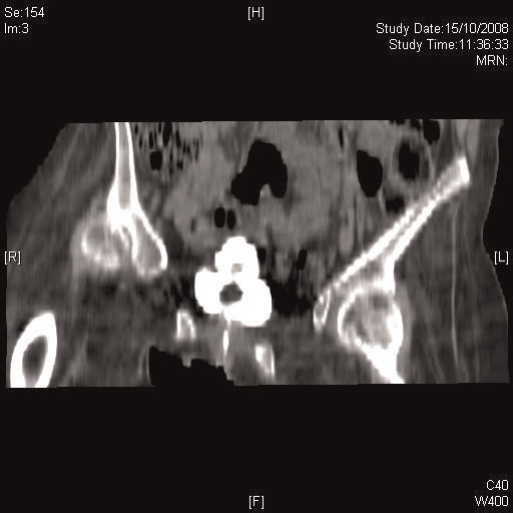
Coronal reconstruction of CT cystography, performed on 15 October 2008, shows hourglass bladder with smaller superior compartment and larger inferior compartment.

**Figure 8.**
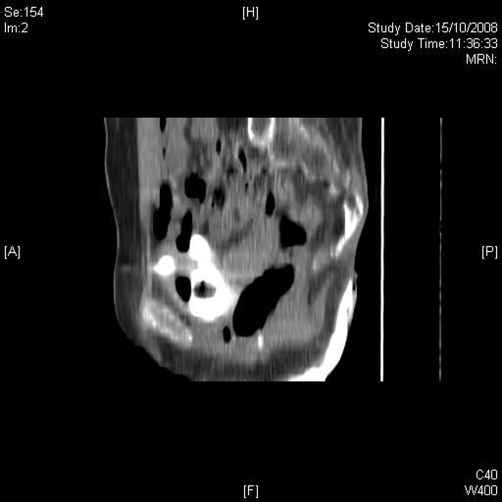
Sagittal reconstruction of CT cystography, performed on 15 October 2008, shows hourglass bladder with narrow waist.

Ultrasound examination was performed on 30 September 2008. The suprapubic catheter tip and balloon were located in upper compartment of hourglass bladder, whereas the urethral catheter was placed in the inferior compartment, which appeared to be the main lumen of bladder (Figure 6). Computed tomography (CT) cystogram was performed on 17 October 2008. Coronal and sagittal multiplanar reconstruction of the images confirmed the hourglass shape of urinary bladder (Figures 7 and 8). The urethral catheter was lying in the lower part of hourglass bladder while the suprapubic catheter was placed in the upper part, which was smaller. When the bladder was distended, the waist of hourglass bladder measured 3 cm.

**Figure 9.**
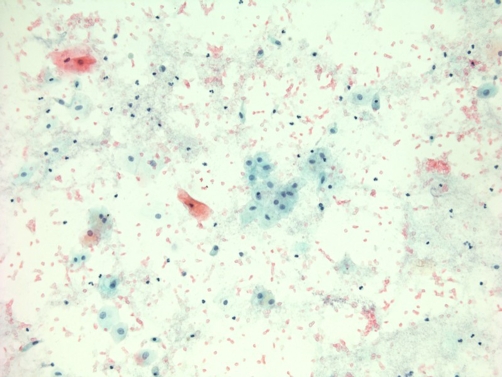
Representative field from urine cytology specimen (Papanicolau stain) showing benign urothelial and squamous cells with numerous erythrocytes and occasional inflammatory cells. No malignant cells were present.

Cytology of urine specimen sent on 11 November 2008, showed benign urothelial and squamous cells with numerous erythrocytes and occasional inflammatory cells. No malignant cells were present (Figure 9).

This patient continued to manage her bladder with suprapubic and urethral catheters. The suprapubic catheter required careful positioning while changing. It was likely that on the occasion when suprapubic catheter failed to drain any urine, the balloon of suprapubic catheter had probably been inflated right at the waist of the hourglass bladder with the tip bent over the inflated balloon.

## Discussion

When a suprapubic catheter is not draining urine, the catheter is flushed with sterile 0.9% sodium chloride. If the catheter is blocked, it is replaced promptly with a new catheter. These measures will restore satisfactory drainage of urine in most cases. If the suprapubic catheter still fails to drain urine, following rare possibilities should be considered.

- The tip of the catheter may be burrowing in to the bladder wall [3].
- The Foley catheter may have been introduced inadvertently in to a vesical diverticulum with the balloon occluding the neck of diverticulum.
- In the past, we have made the mistake of inserting tip of Foley catheter in the proximal urethra through a patulous bladder neck and inflating the balloon in proximal urethra or bladder neck. This happened in a male spinal cord injury patient, who had undergone transurethral resection of bladder neck. The previous bladder neck resection had resulted in a wide bladder neck, which facilitated inadvertent insertion of suprapubic Foley catheter in to the proximal urethra.

When a freshly inserted suprapubic catheter fails to drain urine satisfactorily, we usually perform cystography to check the position of the tip of catheter and the location of Foley balloon. Ultrasound scan and CT cystography provide more information than conventional cystography. In this patient, cystogram revealed hourglass bladder, which was confirmed subsequently by ultrasound scan and CT cystography.

Hourglass deformity of bladder has been described following seromuscular colocystoplasty in the treatment of mixed neurogenic urinary incontinence in children [4], and in patients who underwent bladder autoaugmentation to treat neuropathic bladder secondary to myelomeningocele [5]. Very rarely hourglass bladder may be a congenital deformity [6]. In the case of congenital hourglass bladder, the intravesical lumen is horizontally divided into upper and lower compartments and the bladder looks like an hourglass. Most patients suffering from this disorder are adults. It occurs predominantly in men.

Possible contributory factors for development of hourglass bladder in spinal cord injury patients are: traction applied to dome of urinary bladder by Foley balloon when suprapubic catheter is taped *tightly* to anterior abdominal wall for several months; uncoordinated contractions of detrusor muscle; chronic cystitis leading to hypertrophy of bladder wall. In our patient, the most likely reason for occurrence of hourglass bladder balloon was traction on dome of urinary bladder by suprapubic catheter. The balloon of suprapubic Foley catheter probably acted as a traction device to produce “traction diverticulum” of urinary bladder [7]. Continuous traction of the dome of urinary bladder over a period of many months eventually resulted in hourglass shape of urinary bladder. At present, our patient is happy to manage her bladder with urethral and suprapubic catheters. It is possible that she may require urinary diversion at a future date.

## Conclusions

When prompt replacement of a mal-functioning suprapubic catheter fails to rectify the problem, CT cystography should be performed to check precise location of suprapubic catheter and structural abnormalities of urinary bladder. In this patient, cystogram revealed hourglass bladder. Possible reasons for development of hourglass bladder in spinal cord injury patients are: traction applied to dome of urinary bladder by Foley balloon when suprapubic catheter is taped tightly to anterior abdominal wall for prolonged period; uncoordinated contractions of detrusor muscle; chronic cystitis leading to hypertrophy of bladder wall.

## List of abbreviations

CT: Computer tomography
MRI: Magnetic resonance imaging
